# Hyper-*O*-GlcNAcylation induces cisplatin resistance via regulation of p53 and c-Myc in human lung carcinoma

**DOI:** 10.1038/s41598-017-10886-x

**Published:** 2017-09-06

**Authors:** Sudjit Luanpitpong, Paweorn Angsutararux, Parinya Samart, Nawin Chanthra, Pithi Chanvorachote, Surapol Issaragrisil

**Affiliations:** 1 Siriraj Center of Excellence for Stem Cell Research, Faculty of Medicine Siriraj Hospital, Mahidol University, Bangkok, 10700 Thailand; 2 Department of Immunology, Faculty of Medicine Siriraj Hospital, Mahidol University, Bangkok, 10700 Thailand; 3 Department of Pharmacology and Physiology, Faculty of Pharmaceutical Sciences, Chulalongkorn University, Bangkok, 10330 Thailand; 4 Division of Hematology, Department of Medicine, Faculty of Medicine Siriraj Hospital, Mahidol University, Bangkok, 10700 Thailand; 5 Bangkok Hematology Center, Wattanosoth Hospital, BDMS Center of Excellence for Cancer, Bangkok, 10310 Thailand

## Abstract

Aberrant metabolism in hexosamine biosynthetic pathway (HBP) has been observed in several cancers, affecting cellular signaling and tumor progression. However, the role of *O*-GlcNAcylation, a post-translational modification through HBP flux, in apoptosis remains unclear. Here, we found that hyper*-O*-GlcNAcylation in lung carcinoma cells by *O*-GlcNAcase inhibition renders the cells to apoptosis resistance to cisplatin (CDDP). Profiling of various key regulatory proteins revealed an implication of either p53 or c-Myc in the apoptosis regulation by *O*-GlcNAcylation, independent of p53 status. Using co-immunoprecipitation and correlation analyses, we found that *O*-GlcNAcylation of p53 under certain cellular contexts, i.e. high p53 activation, promotes its ubiquitin-mediated proteasomal degradation, resulting in a gain of oncogenic and anti-apoptotic functions. By contrast, *O*-GlcNAcylation of c-Myc inhibits its ubiquitination and subsequent proteasomal degradation. Gene manipulation studies revealed that *O*-GlcNAcylation of p53/c-Myc is in part a regulator of CDDP-induced apoptosis. Accordingly, we classified CDDP resistance by hyper*-O-*GlcNAcylation in lung carcinoma cells as either p53 or c-Myc dependence based on their molecular targets. Together, our findings provide novel mechanisms for the regulation of lung cancer cell apoptosis that could be important in understanding clinical drug resistance and suggest *O*-GlcNAcylation as a potential target for cancer therapy.

## Introduction

Cancer cells increase nutrient consumption leading to deregulating cellular metabolism, an emerging characteristic hallmark of cancer^1, 2^. The hexosamine biosynthetic pathway utilizes major essential nutrients and metabolic intermediates including glucose, glucosamine, amino acid, fatty acid and nucleotide into an end product of nucleotide sugar uridine diphosphate N-acetyl-glucosamine (UDP-GlcNAc)^3^. *O*-linked-β-*N*-acetylglucosomaine (*O*-GlcNAc) is subsequently transferred from UDP-GlcNAc to serine (Ser) and/or threonine (Thr) residues in substrate proteins by the enzyme *O*-GlcNAc transferase (OGT) causing a post-translational modification (PTM) called *O*-GlcNAcylation. This PTM which can be removed by the enzyme *O*-GlcNAcase (OGA; encoded by *MGEA5*) can alter protein functions and stability directly and indirectly, e.g. by competing with phosphorylation sites. Increasing evidence has shown that PTM dynamic regulates gene transcription and cellular signaling in response to changes in microenvironment triggered by altered nutrients and stress^4, 5^.

In recent years, elevated *O*-GlcNAcylation (hyper-*O*-GlcNAcylation) has been observed in various cancers, including breast, colon, pancreas, liver and lung^6, 7^, and its roles in oncogenesis and tumor progression have been increasingly reported. *O*-GlcNAcylation was found to modulate cell proliferation and anchorage-independent growth of pancreatic cancer cells through NF-κB activation^8^, whilst induce cell invasion and metastasis of breast cancer cells through the modulation of FOXM1 transcription factor^9^. In lung carcinoma, *O*-GlcNAcylation induced tumor growth by increasing glucose metabolic flux through pentose phosphate pathway^10^. However, the role of *O*-GlcNAcylation in apoptosis and detailed mechanisms by which protein-specific *O*-GlcNAcylation contributes to the apoptotic response to chemotherapy remain largely unknown.

Here we present evidence that hyper-*O*-GlcNAcylation by OGA inhibitors renders human lung carcinoma cells to apoptosis resistance induced by cisplatin (CDDP) via two distinct pathways that are either p53- or c-Myc-dependent, depending on cellular context, independent of p53 status. Specifically, *O*-GlcNAcylation of CDDP-induced p53 promotes ubiquitin-mediated proteasomal degradation, causing p53 instability. On the contrary, when *O*-GlcNAcylation of c-Myc occurs, it inhibits c-Myc ubiquitination and degradation. Our findings unveil a previously unknown mechanism for the regulation of lung tumor cell apoptosis that could be important in understanding clinical drug resistance and may have clinical utility for targeted drug therapy of lung and other cancers whose etiology is dependent on *O*-GlcNAcylation.

## Results

### Expression of *O*-GlcNAc cycling enzymes in human lung carcinoma

**Figure 1:**
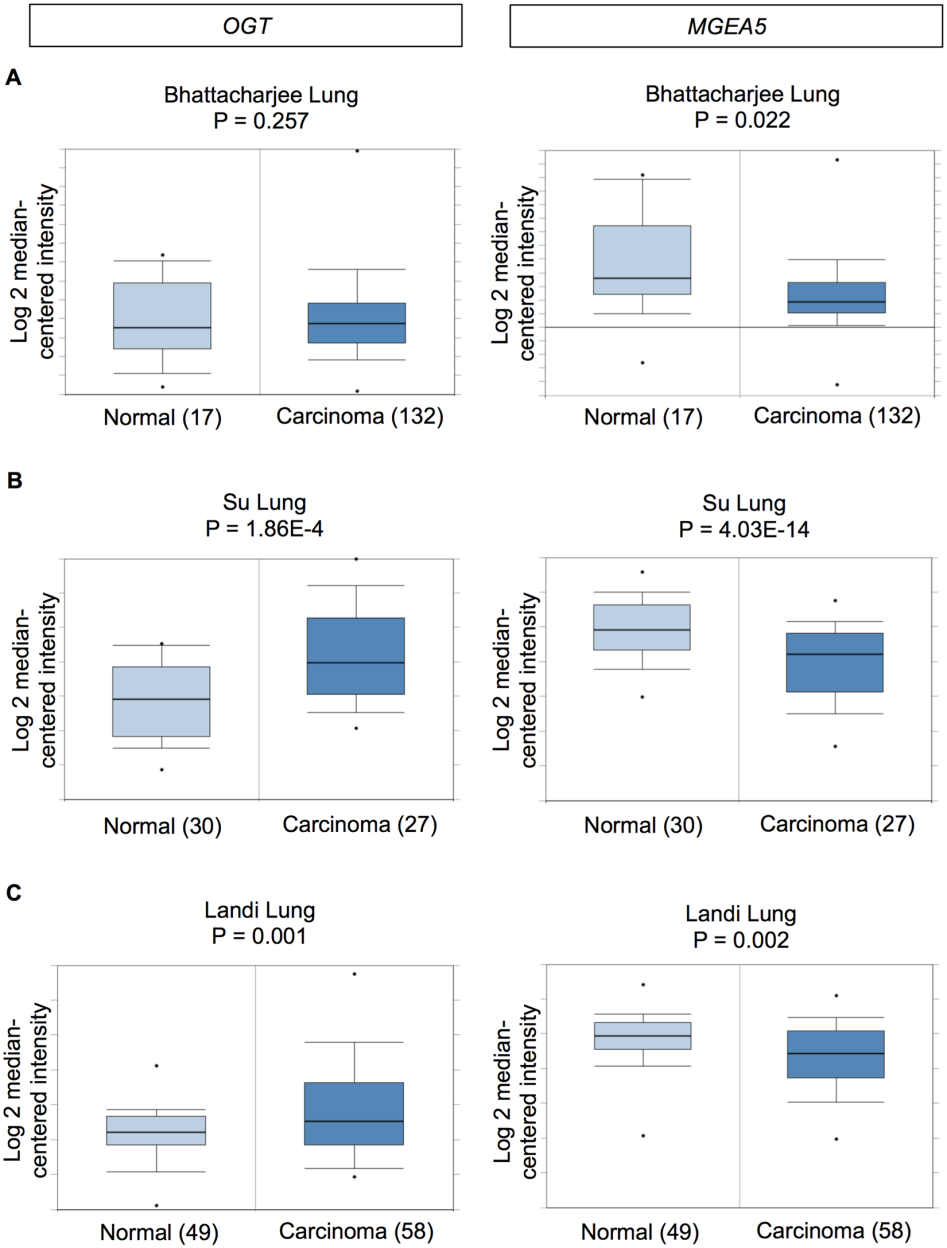
mRNA expression of *O*-GlcNAc cycling enzymes *OGT* and *MGEA5* in clinical lung carcinoma samples in comparison to normal lung tissues on Oncomine^TM^ bioinformatics database. (**A**,**B**,**C**) Differential expression of *OGT* and *MGEA5* (encoded OGA) in the Bhattacharjee Lung, Su Lung and Landi Lung datasets.

Hyper-*O*-GlcNAcylation might be attributable to high OGT and/or low OGA levels. We performed mRNA expression analysis of *OGT* and *MGEA5* (encoded OGA) in human clinical specimens available on Oncomine^TM^ bioinformatics database. An elevated *OGT* expression was observed in the majority of human lung adenocarcinoma tissues compared with normal lung tissues in 7 out of 8 analyzed datasets − one remaining dataset (Bhattachajee) revealed a remarkable decrease in *MGEA5* expression (Fig. 1A). Lung carcinoma tissues in Su, Okayama and Landi datasets were found to have both high *OGT* and low *MGEA5* levels as compared with normal tissues (Fig. 1B,C). These analyses support the previous finding on hyper-*O*-GlcNAcylation in lung carcinoma^11^.

### Hyper-*O*-GlcNAcylation renders cisplatin resistance in human lung carcinoma

**Figure 2:**
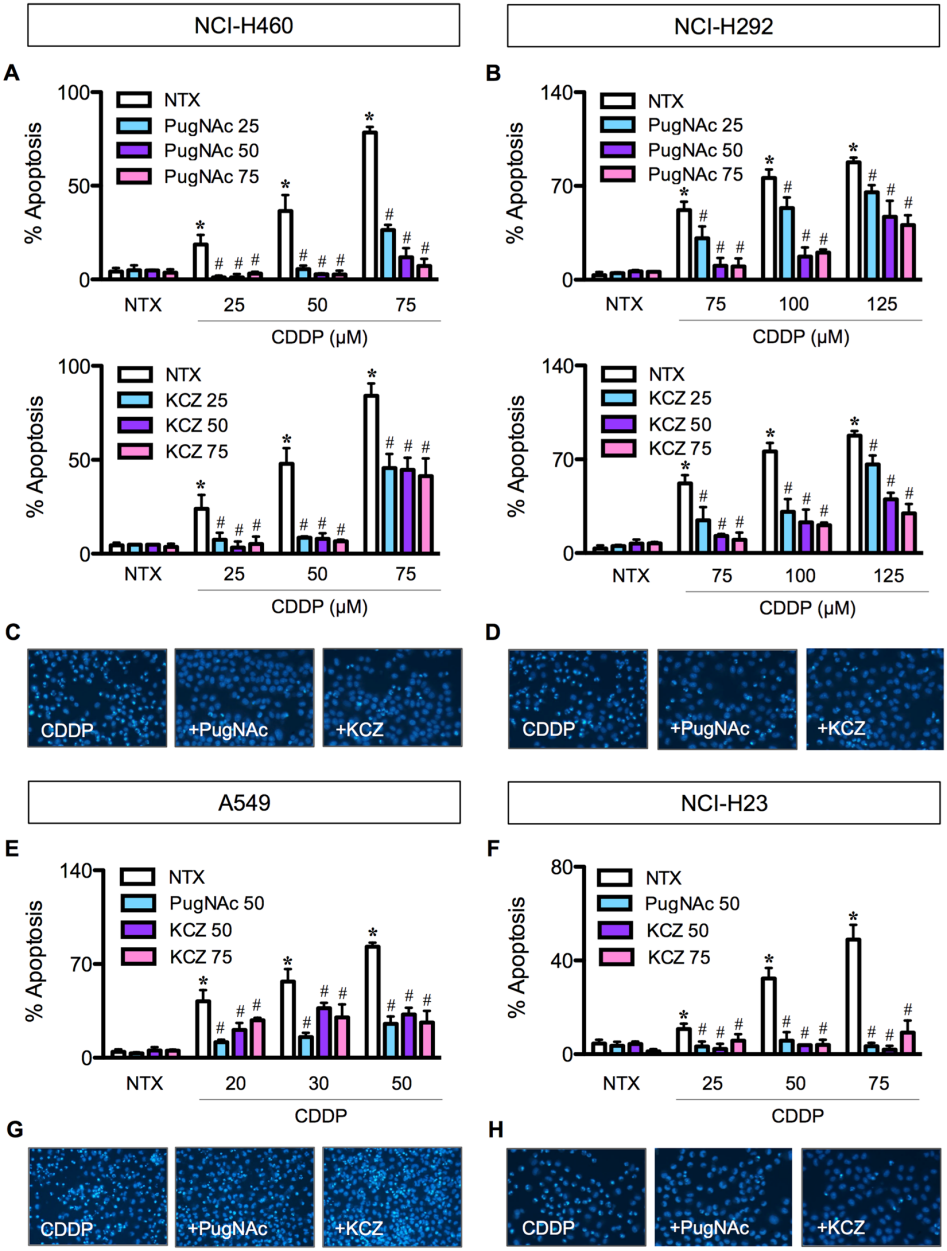
*O*-GlcNAcase inhibitors prevent cisplatin-induced apoptosis in lung carcinoma cells. Multiple human lung carcinoma cell lines, including NCI-H460 (**A**), NCI-H292 (**B**), A549 (**E)** and NCI-H23 (**F**) cells were treated with cisplatin (CDDP; 0–75 μM in NCI-H460 and NCI-H23, 0–125 μM in NCI-H292, and 0–50 μM in A549 cells) for 24 in the presence or absence of *O*-GlcNAcase inhibitors, including PugNAc (25–75 μM) (*upper*) and ketoconazole (KCZ; 25–75 μM) (*lower*), before analysis for apoptosis by Hoechst 33342 assay. (**C**,**D**,**G**,**H**) Fluorescence micrographs of the treated cells stained with Hoechst dye. Plots are means ± S.D. (*n* = 4). *p < 0.05 versus non-treated control. ^#^ *p* < 0.05 versus CDDP-treated cells.

To investigate the role of *O*-GlcNAcylation in apoptosis of human lung carcinoma, we treated a panel of National Cancer Institute (NCI) lung cancer cell lines, including NCI-H460, NCI-H292, NCI-H23 and A549 cells, with a known apoptosis inducer CDDP in the presence or absence of small molecule inhibitors of OGA (removal of *O*-GlcNAc) that cause an increase in global *O*-GlcNAcylation, including PugNAc and KCZ. After a 24 h post-treatment, cell apoptosis was determined by Hoechst 33342 assay. CDDP treatment (0–50 μM in A549; 0–75 μM in NCI-H460 and NCI-H23; and 0–125 μM in NCI-H292 cells) caused a dose-dependent increase in apoptosis in all tested cell lines, the effect that was inhibited by co-treatment of the cells with OGA inhibitors PugNAc (0–75 μM) and KCZ (0–75 μM) (Fig. 2A,B,E and F). The apoptotic cells exhibited condensed and/or fragmented nuclei with intense nuclear fluorescence (Fig. 2C,D,G and H). The inhibitory effect of OGA inhibitors suggests that hyper-*O*-GlcNAcylation plays a critical role in the protection against CDDP-induced apoptosis, which is confirmed by subsequent cell diameter and DNA content analyses (Supplementary Figure S1). As KCZ displays a higher selectivity for human OGA over its closely related lysosomal hexosamidases (HexA/B) than PugNAc^12^, we used KCZ in subsequent molecular studies.

**Figure 3:**
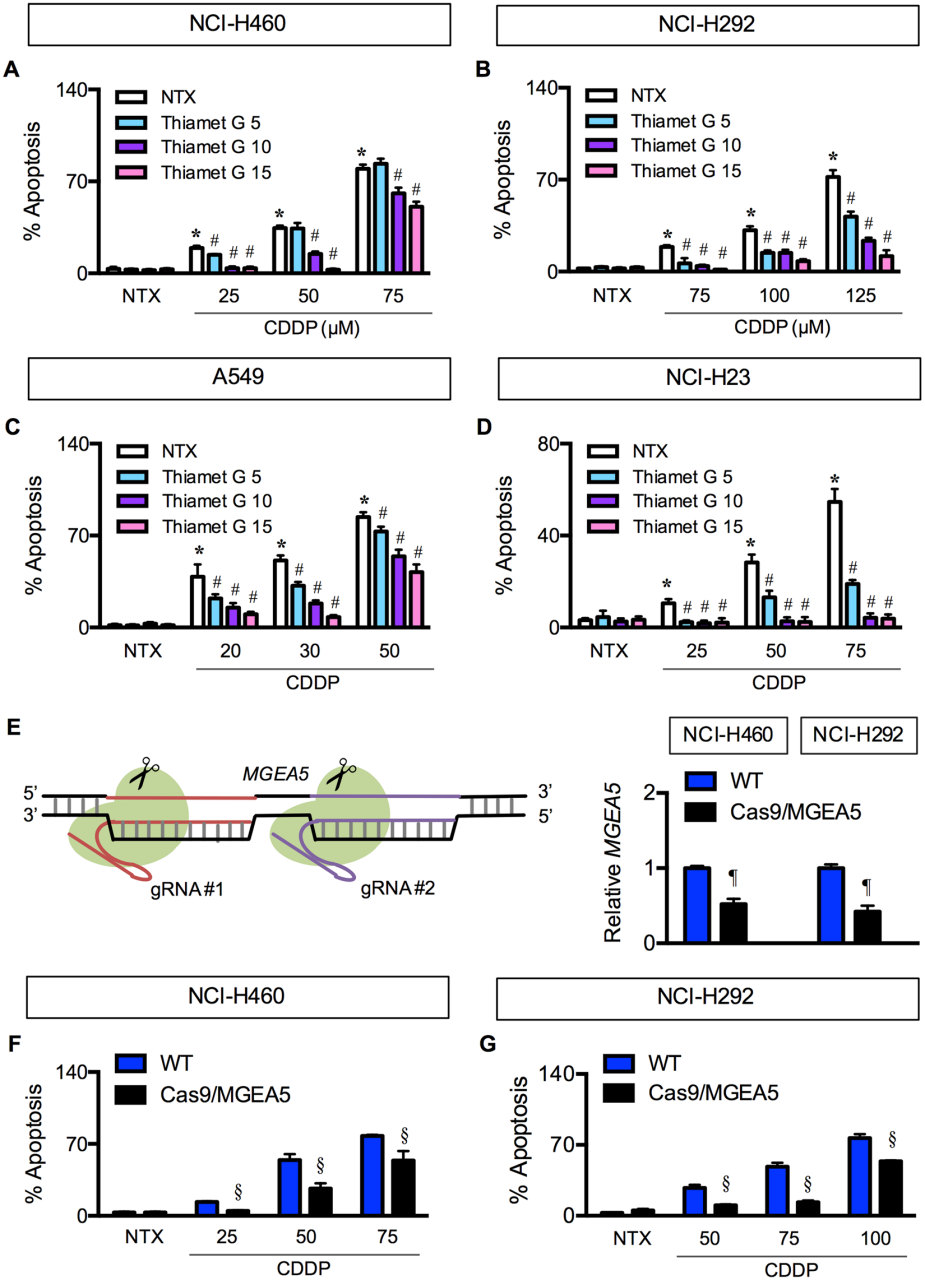
Validation of apoptosis blockage by *O*-GlcNAcase inhibition and repression. (**A**–**D**) Multiple human lung carcinoma cell lines, including NCI-H460 (**A**), NCI-H292 (B), A549 (**C**) and NCI-H23 (**D**) cells were treated with cisplatin (CDDP; 0–75 μM in NCI-H460 and NCI-H23, 0–125 μM in NCI-H292, and 0–50 μM in A549 cells) for 24 h in the presence or absence of a potent and selective *O*-GlcNAcase inhibitor thiamet G (5–15 μM). Apoptosis was determined by Hoechst 33342 assay at 24 h post-treatment. Plots are means ± S.D. (n = 3). *p < 0.05 versus non-treated control. ^#^ *p* < 0.05 versus CDDP-treated cells. (**E**) (*left*) A schematic diagram for a transcriptional repression of *MGEA5* (encoding OGA) using CRISPR/Cas9 system. (*right*) Quantitative real-time PCR of *MGEA5* mRNA expression in NCI-H460 and NCI-H292 cells. Plots are means ± S.D. (n = 3). *p* < 0.05 versus control (WT) cells. (**F**) Effect of *MGEA5* repression on CDDP-induced apoptosis. OGA-knockdown (Cas9/MGEA5) and control (WT) cells were treated with CDDP for 24 h and analyzed for apoptosis using Hoechst 33342 assay. Plots are means ± S.D. (n = 3). ^*§*^ *p* < 0.05 versus cisplatin-treated control (WT) cells.

To strengthen the role of hyper-*O*-GlcNAcylation in apoptosis inhibition and CDDP resistance, we further inhibited or repressed cellular OGA by a novel selective inhibitor thiamet G or by genetic manipulation using CRISPR/Cas9 system. Figure 3A–D shows that apoptosis induction by CDDP was substantially reduced after co-treatment of the cells with thiamet G. This effect was observed in all four cell lines tested and in a dose-dependent manner. For CRISPR-mediated repression of *MGEA5* (encoded OGA), we used two *MGEA5*-targeting guide RNAs (gRNA #1 and #2) to create double-stranded breaks near the promoter region, which resulted in a decrease in *MGEA5* expression in the cell population (Fig. 3E). The OGA-knockdown (Cas9/MGEA5) cells were significantly less responsive to CDDP when compared to control (WT) cells (Fig. 3F,G), thus confirming the inhibitory effect of hyper-*O*-GlcNAcylation on CDDP-induced apoptosis.

### *O*-GlcNAcylation suppresses cisplatin-induced apoptosis through a caspase-dependent pathway

**Figure 4:**
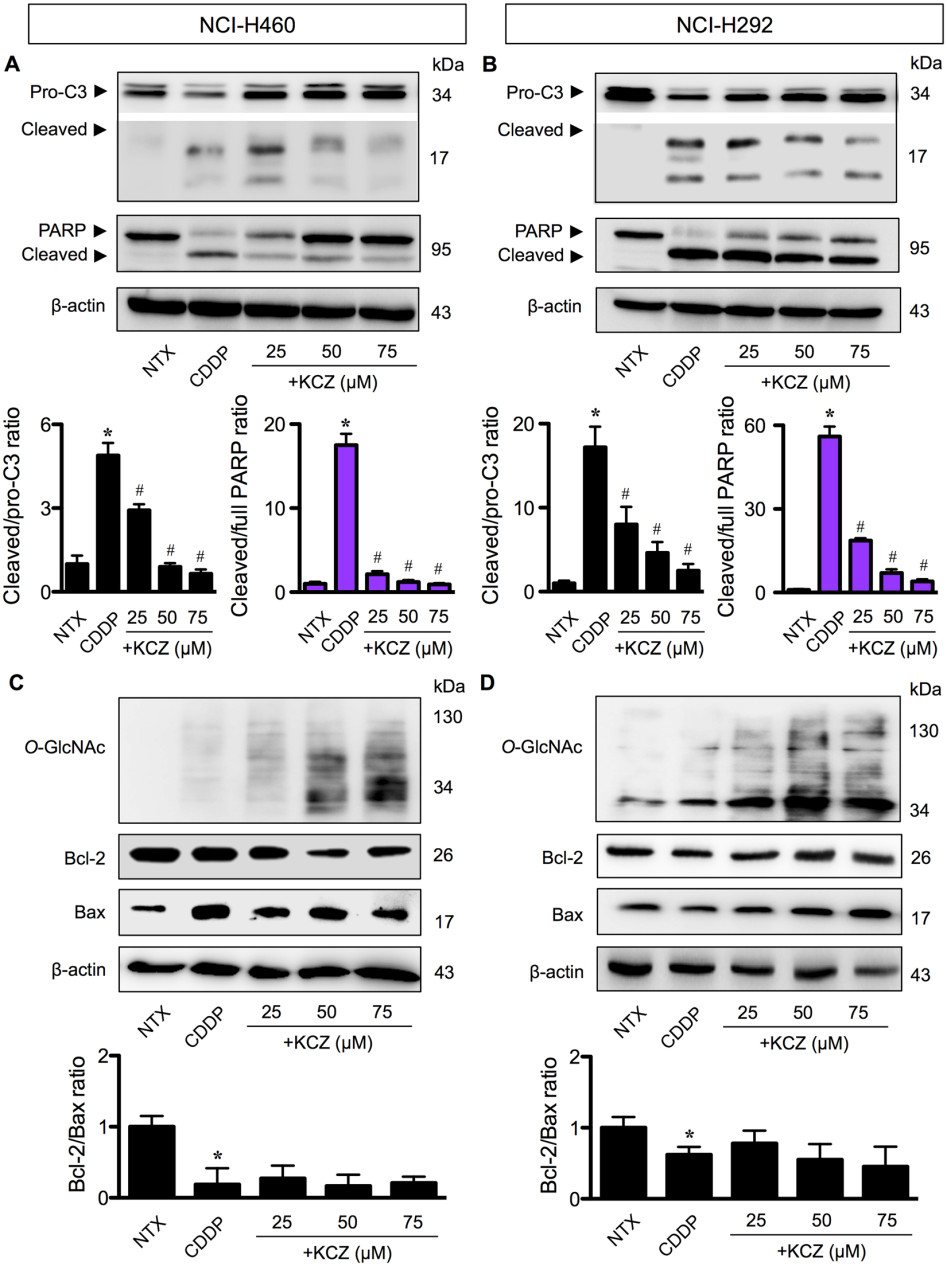
*O*-GlcNAcylation had minimal effect on Bcl-2 and Bax imbalance caused by cisplatin. (**A**,**B**) Human lung carcinoma NCI-H460 and NCI-H292 cells were co-treated with cisplatin (CDDP; 50 or 75 μM) and a highly selective *O*-GlcNAcase inhibitor ketoconazole (KCZ; 25–75 μM) for 24 h and cell lysates were prepared and analyzed for cleaved (active) caspase-3 (C3) and cleaved PARP by Western blotting. Blots were reprobed with anti-β-actin antibody to confirm equal loading of the samples. Immunoblot signals were quantified by densitometry and mean data from three independent experiments (one of which is shown here) were normalized to the loading control, calculated as the fold difference relative to non-treated control and presented as cleaved/pro-C3 and cleaved/full PARP ratios. Cleaved and pro-C3 were cropped from different parts of the same gel and exposure was adjusted to aid the visualization of cleaved C3. (**C**,**D**) Cells were similarly treated with CDDP and KCZ, and Bcl-2 and Bax protein levels were evaluated along with global *O*-GlcNAcylation by Western blotting. Quantitative analysis of Bcl-2 and Bax by densitometry is shown as Bcl-2/Bax ratio. Plots are means ± S.D. (*n* = 3). *p < 0.05 versus non-treated control. ^#^ *p* < 0.05 versus CDDP-treated cells.

To understand how *O*-GlcNAcylation regulates apoptosis, we first characterized the pathway of apoptosis in response to CDDP and KCZ co-treatment. NCI-H460 and NCI-H292 cells were treated with CDDP (50 or 75 μM) alone or in combination with increasing concentrations of KCZ (25–75 μM) in the presence or absence of caspase inhibitor z-DEVD-fmk (10 μM). KCZ was found to suppress caspase-3 activity, the central execution event of apoptosis^13^, in a dose-dependent manner (Supplementary Figure S2) in concomitant with the observed nuclear morphological changes (Fig. 2). The caspase inhibitory effect of KCZ was similar to and synergistic with that of z-DEVD-fmk, suggesting that *O*-GlcNAcylation inhibits apoptosis induced by CDDP through caspase inhibition. Western blot analysis further confirmed the inhibitory effect of KCZ on caspase-3 activation and its downstream event PARP cleavage (Fig. 4A,B), thus substantiating that *O*-GlcNAcylation prevents caspase-dependent apoptosis.

Bcl-2 family proteins regulate apoptosis along the intrinsic mitochondrial apoptosis pathway that is activated in response to various apoptotic stimuli, including CDDP^14, 15^. To determine whether Bcl-2 family proteins involve in the inhibitory effect of *O*-GlcNAcylation on caspase activation and apoptosis, we co-treated the cells with CDDP and KCZ and analyzed for Bcl-2 and Bax expression in correlation to hyper*-O*-GlcNAcylation by Western blotting. Figure 4C,D shows that although CDDP was able to tilt the balance between pro-apoptotic Bax and anti-apoptotic Bcl-2 in favor of apoptosis, i.e. a decrease in Bcl-2/Bax ratio, KCZ did not exert its effect through the Bcl-2/Bax balance since we observed no significant changes in the ratio after co-treating the cells with KCZ compared to CDDP treatment alone. Further analysis of other Bcl-2 family members revealed insignificant changes of anti-apoptosis proteins (e.g. Bcl-xL and Mcl-1) and pro-apoptotic proteins (Bak and Bid) in relation to the apoptotic responses (Supplementary Figure S3).

### Distinct effects of *O*-GlcNAcylation on p53 and c-Myc in wild-type and mutant p53 lung cancer cells

**Figure 5:**
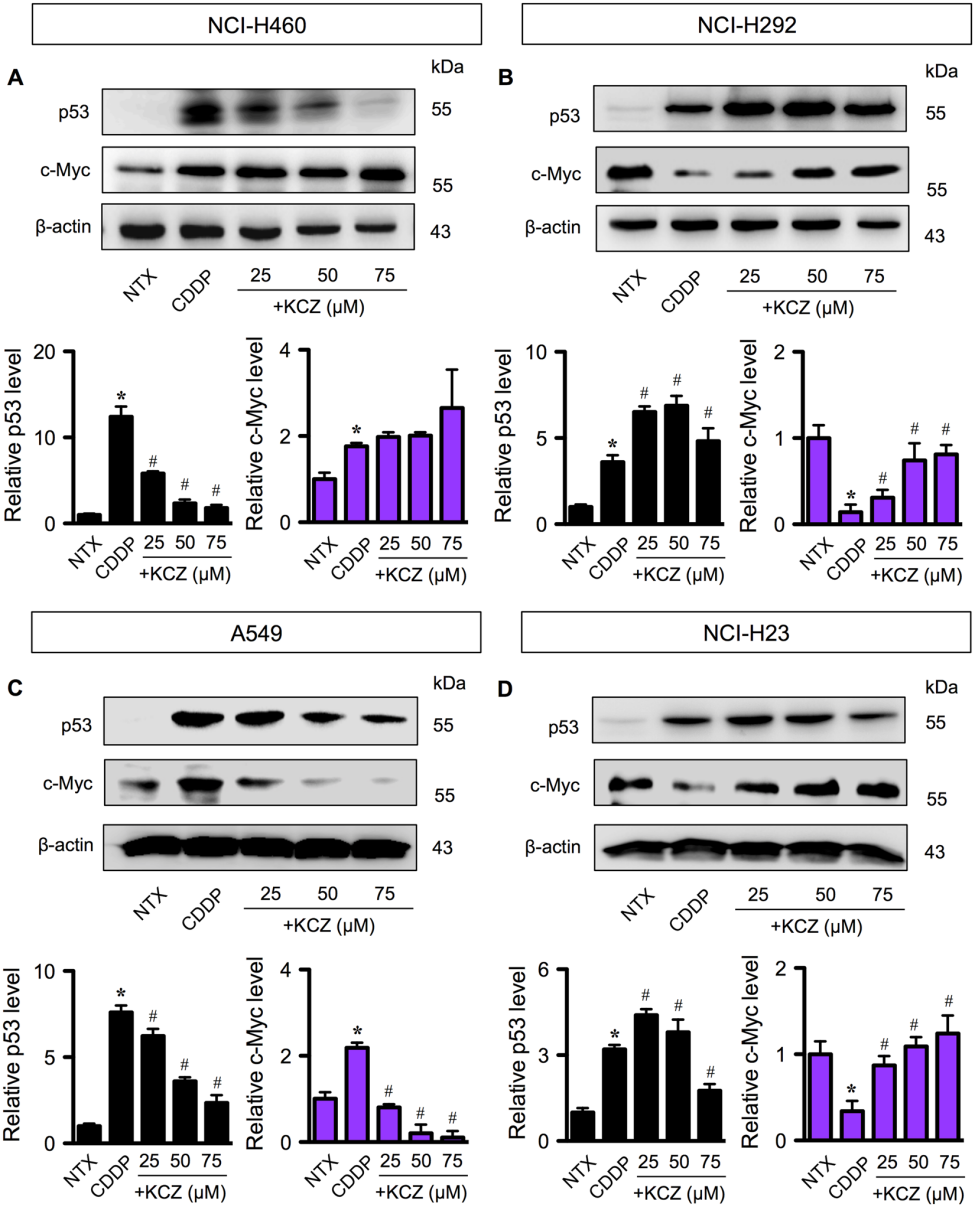
*O*-GlcNAcylation targets p53 or c-Myc in cisplatin-induced apoptosis independent of p53 status. Multiple human lung carcinoma cell lines, including NCI-H460 (**A**), NCI-H292 (**B**), A549, (**C**) and NCI-H23 (**D**) cells were treated with cisplatin (CDDP; 50 μM in NCI-H460 and NCI-H23, 30 μM in A549, and 100 μM in NCI-H292 cells) for 24 in the presence or absence of increasing concentrations of *O*-GlcNAcase inhibitor ketoconazole (KCZ; 25–75 μM). Cell lysates were then prepared and analyzed for p53 and c-Myc by Western blotting. Plots are means ± S.D. (*n* = 3). *p < 0.05 versus non-treated control. ^#^ *p* < 0.05 versus CDDP-treated cells.

Having demonstrated that *O-*GlcNAcylation has minimal effects on the regulation and/or expression of critical Bcl-2 family proteins, we next tested its effect on the central oncogenic switch of c-Myc oncogene and p53 tumor suppressor, both of which are known targets of *O-*GlcNAcylation^16−19^. Figure 5A–D shows that although CDDP induced p53 activation in all wild-type (NCI-H460, NCI-H292 and A549)^20, 21^ and mutant p53 (NCI-H23) cells^22^, the level of activation differs substantially among these cells independent of their p53 status. We also observed that CDDP unexpectedly induced c-Myc in the cells with high p53 activation (>5-fold), i.e. NCI-H460 and A549 cells, but reduced c-Myc in the cells with low p53 activation (<5-fold), i.e. NCI-H292 and NCI-H23 cells.

We further found that KCZ dose-dependently rendered CDDP-induced p53 activation only in NCI-H460 and A549 cells where high p53 activation was detected, but not in NCI-H292 and NCI-H23 cells. In cells with low p53 activity, the protective effect of KCZ on CDDP-induced apoptosis appears to be dependent on c-Myc level based on the observations that (i) KCZ hampered CDDP-driven c-Myc reduction in concomitant with the increase in cell survival in a dose-dependent manner, and (ii) KCZ had minimal effect on p53 protein level, suggesting that the target proteins of *O-*GlcNAcylation may vary depending on cellular context, i.e. the presence of active p53.

### Cisplatin resistance by hyper-*O-*GlcNAcylation is regulated by p53 or c-Myc

**Figure 6:**
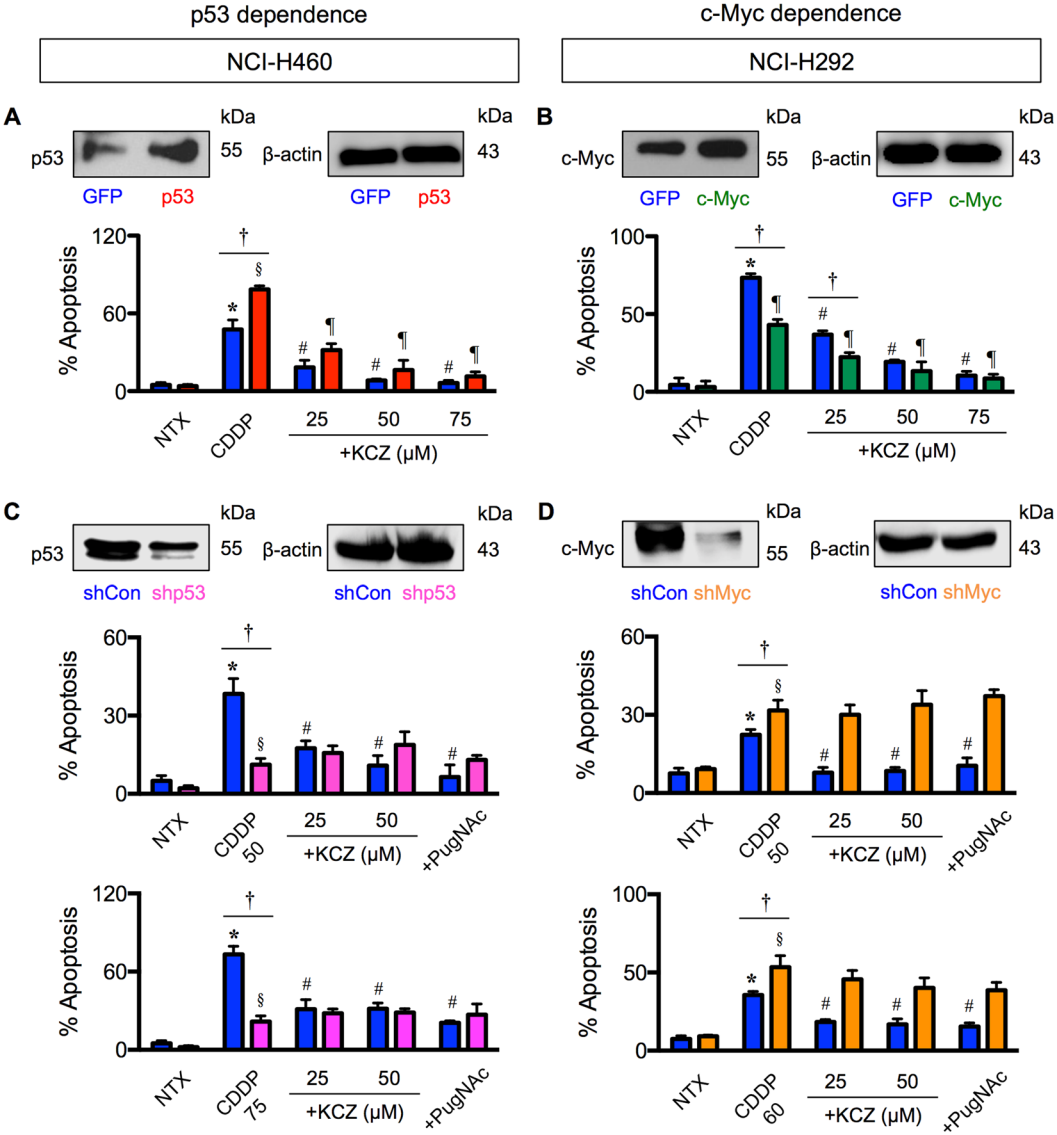
p53/c-Myc is crucial for *O*-GlcNAc-mediated cisplatin resistance. (**A**,**B**) Human lung carcinoma NCI-H460 and NCI-H292 cells were transfected with p53 or c-Myc expression plasmid, co-treated with cisplatin (CDDP; 50 μM in NCI-H460 and 100 μM in NCI-H292 cells) and ketoconazole (KCZ; 25–75 μM), and analyzed for apoptosis by Hoechst 33342 assay at 24 h. (**C**,**D**) p53 and c-Myc knockdown experiments were performed using NCI-H460 or NCI-H292 cells treated with retroviral particles carrying shp53 or with lentiviral particles carrying shMyc. Cells were co-treated CDDP (50–75 μM in NCI-H460 and 50–60 μM in NCI-H292 cells) and KCZ (25–50 μM), and similarly analyzed for apoptosis at 24 h post-treatment. Plots are means ± S.D. (*n* = 3). *^,*§*^ *p* < 0.05 versus non-treated GFP or p53/c-Myc-overexpressed cells and non-treated shCon or shp53/shMyc cells. ^#^ *p* < 0.05 versus CDDP-treated GFP or p53/c-Myc-overexpressed cells and CDDP-treated shCon cells. ^*†*^ *p* < 0.05 versus CDDP-treated GFP cells and CDDP-treated shp53/shMyc cells.

To test whether *O-*GlcNAcylation of p53/c-Myc is a potential regulator of CDDP-induced apoptosis in lung cancer cells under specified context, NCI-H460 and NCI-H292 cells were ectopically transfected with p53 and c-Myc, respectively, and apoptosis in response to CDDP and KCZ co-treatment was determined. Figure 6A shows that p53-overexpressing NCI-H460 cells were more susceptible to CDDP-induced apoptosis than control transfected cells, yet they were protected by KCZ. On the other hand, c-Myc-overexpressing NCI-H292 cells were less susceptible to CDDP-induced apoptosis and had strengthening protective effect of KCZ compared to control cells (Fig. 6B). The effects of p53 and c-Myc overexpression in A549 and NCI-H23 cells are shown in Supplementary Figure S4.

To verify that p53/c-Myc is required for *O-*GlcNAc-mediated cisplatin resistance, p53 and c-Myc expression were inhibited by RNA interference using short hairpin RNAs against *TP53* (shp53) and *MYC* (shMyc) in NCI-H460 and NCI-H292 cells, and their effects on apoptosis inhibition by OGA inhibitor were examined. Figure 6C,D shows that knockdown of p53 rendered NCI-H460 cells to CDDP resistance, while knockdown of c-Myc sensitized NCI-H292 cells to CDDP. KCZ noticeably failed to protect cells from CDDP-induced apoptosis in both NCI-H460-shp53 cells and NCI-H292-shMyc cells, the results that were confirmed by another OGA inhibitor PugNAc, indicating that p53/c-Myc is critical for the apoptosis inhibition by *O-*GlcNAcylation. Altogether, these results indicate that CDDP-induced apoptosis was regulated in part by *O-*GlcNAcylation of p53 or c-Myc. Accordingly, we classified CDDP resistance by hyper-*O-*GlcNAcylation in lung carcinoma cells as either p53 or c-Myc dependence based on the target of *O-*GlcNAcylation.

### Effects of *O*-GlcNAcylation on ubiquitination of p53 and c-Myc

**Figure 7:**
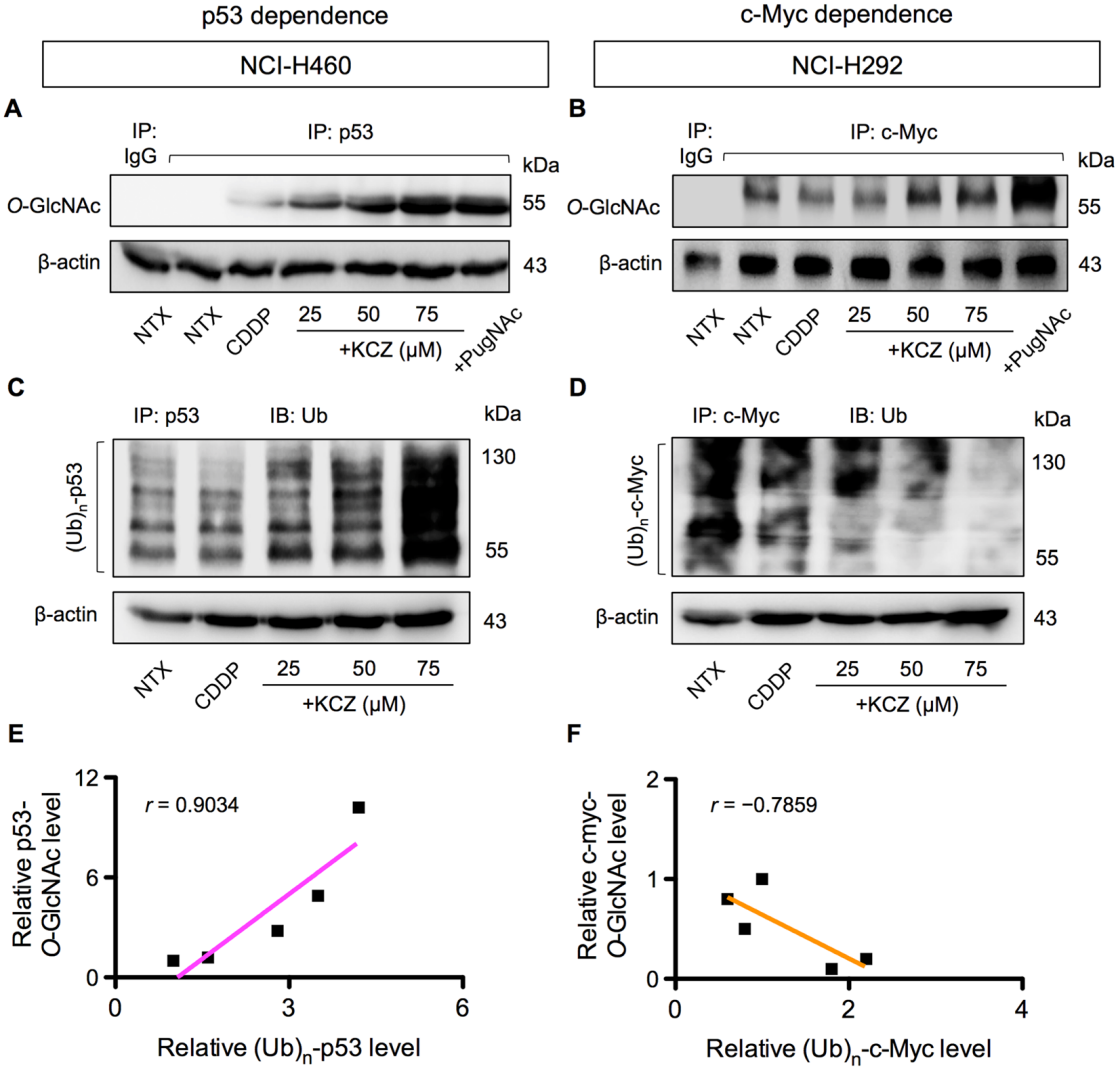
*O*-GlcNAcylation of p53 and c-Myc interferes with their ubiquitin-proteasomal degradation in cisplatin-treated cells. NCI-H460 cells and NCI-H292 cells were treated with CDDP and KCZ for 3 h, and cell lysates were prepared and immunoprecipitated using anti-p53 and anti-c-Myc antibodies. The immune complexes were analyzed for *O*-GlcNAcylation (**A**,**B**) or ubiquitination (Ub) (**C**,**D**) by Western blotting. Immunoblots were performed on cell lysates served as IP input using anti-β-actin antibody to confirm equal loading of the samples. (**E**,**F**) Correlation analysis of *O*-GlcNAcylation and ubiquitination of p53 and c-Myc in CDDP-treated cells in the presence or absence of KCZ.

PTMs are the key mechanism that diversifies the proteome complexity and are known to influence protein stability and function^23, 24^. Ubiquitination is a major PTM that occurs on Ser and/or Thr residues and controls protein degradation and stability via ubiquitin-proteasomal pathway^25^. As *O*-GlcNAcylation similarly occurs on Ser and/or Thr residues of protein, it is conceivable that *O-*GlcNAcylation of p53 and c-Myc might interfere with their ubiquitination and subsequent expression. NCI-H460 and NCI-H292 cells were treated with CDDP and KCZ, and p53 or c-Myc was immunoprecipitated and subjected to Western blot analysis using an *O*-GlcNAc-specific antibody (RL2). Figure 7A,B shows that while CDDP had a minimal effect on *O*-GlcNAcylation of p53 in NCI-H460 cells and of c-Myc in NCI-H292 cells, KCZ indeed stimulated hyper-*O*-GlcNAcylation of p53 and c-Myc. Similar results were observed when the cells were treated with another OGA inhibitor, PugNAc, thus confirming the inductive effect of OGA inhibitor on *O*-GlcNAcylation of p53 and c-Myc. Ubiquitination of p53 and c-Myc was further analyzed after the cells were similarly exposed to CDDP and KCZ. Figure 7C shows that KCZ significantly increased p53 ubiquitination in NCI-H460 cells in a dose-dependent manner in parallel with the observed increase in p53 *O*-GlcNAcylation (Fig. 7A) and the decrease in its expression (Fig. 5A). Further analysis of the correlation between p53 *O*-GlcNAcylation and ubiquitination revealed the positive relationship of the two events with a correlation coefficient (*r*) of 0.9034 (Fig. 7E), supporting the notion that ubiquitin-mediated p53 degradation was strengthen by its *O*-GlcNAcylation. By contrast, KCZ remarkably decreased c-Myc ubiquitination in NCI-H292 cells in concomitant with the observed decrease in *O*-GlcNAcylation (Fig. 7D), an inverse relationship with an *r* value of −0.7859 (Fig. 7F), and with the increase in its expression (Fig. 5B), thus substantiating the interfering effect of *O*-GlcNAcylation on c-Myc ubiquitination. Taken together, our findings support that *O*-GlcNAcylation of certain target proteins, e.g. p53 and c-Myc, interferes with their ubiquitination, affecting protein stability and apoptotic responses of lung carcinoma cells to chemotherapy.

## Discussion

Lung cancer is one of the leading causes of cancer-related death worldwide, partly due to an innate and acquired resistance to chemotherapy that critically limits the outcome of treatments^26, 27^. Comprehensive understanding of the molecular basis of apoptosis resistance would facilitate in the identification of potential novel therapeutic targets. The present study identifies the previously unknown role of hyper-*O*-GlcNAcylation in lung carcinoma cells in regulating a distinct set of target proteins that involve in apoptosis resistance to CDDP, the frontline chemotherapy for both non-small cell lung carcinoma (NSCLC) and small cell lung carcinoma (SCLC)^28, 29^.

Reprogramming in glucose metabolism and energy production in cancers has been substantiated for decades, known as the Warburg effect, in which cancer cells increase glucose uptake and rely preferentially on aerobic glycolysis instead of oxidative phosphorylation for ATP production, even under normoxic conditions^30, 31^. The HBP is a branch of glucose metabolism that consumes approximately 5% of total glucose, and thus the abundance of glucose in cancer cells may also drive this pathway and ultimately elevate *O*-GlcNAcylation. Immunohistochemical analysis of tissue microarrays revealed an increase in global *O*-GlcNAcylation in lung cancer tissues compared with their corresponding normal adjacent tissues^11^. Additionally, we analyzed the mRNA expression of *OGT* and *MGEA5* (encoded OGA) using Oncomine^TM^ bioinformatics database and found a remarkable increase in the *OGT* and/or a decrease in the *MGEA5* in lung carcinoma tissues compared with normal lung tissues in many datasets (Fig. 1).

To investigate the role of *O*-GlcNAcylation in the apoptosis response of lung carcinoma to CDDP, we used small molecule inhibitors of OGA and CRISPR-mediated repression of *MGEA5* to elevate the level of global *O*-GlcNAcylation. We observed that in all lung carcinoma cell lines examined hyper*-O*-GlcNAcylation protects the cells from CDDP-induced apoptosis (Figs 2 and 3). To address the potential mechanisms of how *O*-GlcNAcylation rendered lung carcinoma cells to apoptosis resistance, we first identified the changes in apoptosis-regulatory proteins during hyper*-O*-GlcNAcylation. We found that although CDDP disrupted the balance of Bcl-2/Bax ratio in favor of apoptosis, hyper*-O*-GlcNAcylation had minimal effect on the disrupted balance (Fig. 4), indicating that *O*-GlcNAcylation did not protect against CDDP-induced apoptosis through the Bcl-2/Bax axis.

**Figure 8:**
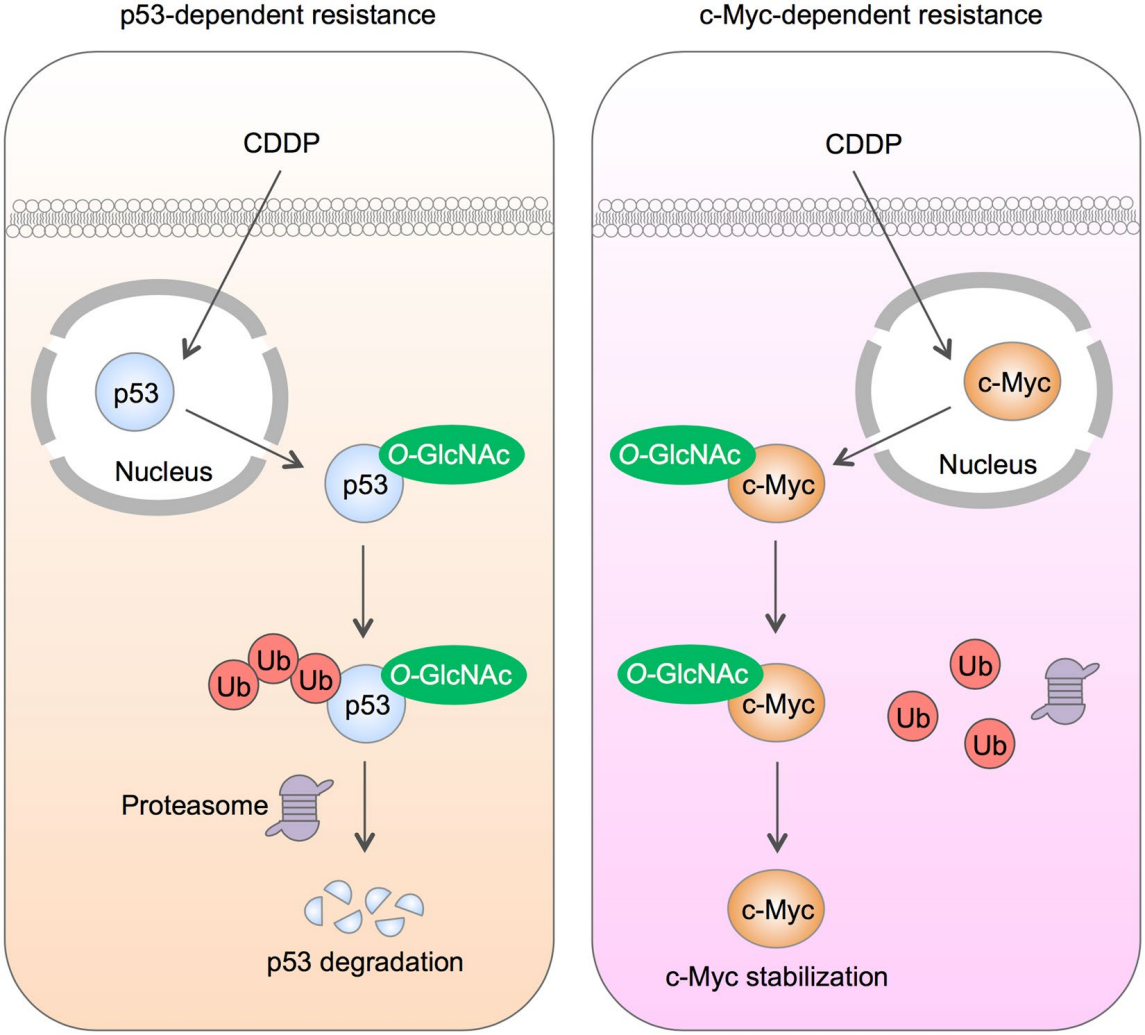
A schematic working model for the mechanisms of cisplatin resistance mediated by *O*-GlcNAcylation. Depending on the cellular context, *O*-GlcNAcylation targets either p53 or c-Myc in cisplatin-treated cells and interferes with its ubiquitin-mediated proteasomal degradation that causes either p53 degradation or c-Myc stabilization.

Aberrant p53 tumor suppressor and/or c-Myc oncogene expression is a common feature in the majority of human cancers. p53 mutations, i.e. missense mutations, frameshift insertions and deletions, and point mutations, were reported to occur in approximately 50% of NSCLC and 80% of SCLC^32, 33^, while c-Myc mutations, i.e. DNA amplification overexpression, were more common in SCLC in up to 40% of all cases^33^. Here, we found that hyper-*O*-GlcNAcylation could render lung carcinoma cells to apoptosis resistance through distinct mechanisms that involve p53 or c-Myc, depending on cellular context, i.e. the presence of active p53. In lung carcinoma cells with high CDDP-induced p53 activation, hyper-*O*-GlcNAcylation targets p53, promotes its ubiquitination and subsequent p53 degradation, resulting in the gain of oncogenic and anti-apoptotic functions (Figs 5–7). By contrast in lung carcinoma cells with low p53 activation, hyper-*O*-GlcNAcylation has minimal effect on p53 and instead regulates c-Myc stability by interfering with its ubiquitin-mediated degradation. These notions are supported by the correlation analysis between *O*-GlcNAcylation and ubiquitination of p53 or c-Myc. Based on the observed findings, we proposed two distinct mechanisms of CDDP resistance in lung carcinoma that are either p53 dependence or c-Myc dependence, as schematically summarized in Fig. 8. It is worth noting that our finding on the induction of p53 degradation by hyper-*O*-GlcNAcylation is inconsistent with a previous study in breast cancer cells that reported the stabilization of p53 by *O*-GlcNAcylation at Ser 149 through the weakening of phosphorylation at Thr 155^18^. We postulate that this discrepancy may be due to the distinct effect of site-specific *O*-GlcNAcylation that is supported by the previous reports showing: (i) p53 contains multiple sites for *O*-GlcNAcylation^18^; and (ii) the major target of CDDP-induced phosphorylation is Ser 15^34, 35^.

In conclusion, our findings unveil a novel mechanism of chemoresistance in lung cancer cells that involves *O*-GlcNAcylation of p53 and c-Myc, which in turn interferes with their ubiquitin-proteasomal degradation. As hyper-*O*-GlcNAcylation is one of the initial events for lung cancer cells to escape from apoptosis, suppression of this PTM may serve as a novel therapeutic strategy for resistant lung cancers.

## Materials and Methods

### Oncomine^TM^ bioinformatics database analysis

mRNA expression of *OGT* and *MGEA5* in lung adenocarcinoma tissues were analyzed in comparison to normal lung tissues from 8 available datasets in Oncomine^TM^ bioinformatics database (https://www.oncomine.org/resource/login.html). The reporter ID (#) and platform for each analyzed dataset were as follows: Bhattachajee Lung #38614_s_at on Human Genome U95A-Av2 Array; Garber Lung #IMAGE:143790 (not Oncomine^TM^ pre-defined platform); Hou Lung 207563_s_at on Human Genome U133 Plus 2.0 Array; Landi Lung #207563_s_at on Human Genome U133A Array; Okayama Lung #207563_s_at on Human Genome U133 Plus 2.0 Array; Selamat Lung #ILMN_1697639 on Illumina HumanWG-6 v3.0 Expression Beadchip; Stearman Lung #38614_s_at on Human Genome U95A-Av2 Array; and Su Lung #207563_s_at on Human Genome U133A Array. The P value for statistical significance was set up as 0.05, while the fold change was defined as all.

### Cell culture

Human lung carcinoma cell lines, including NCI-H460, NCI-H292, NCI-H23 and A549 cells, were obtained from American Type Culture Collection (ATCC; Manassas, VA). A549 cells were cultured in DMEM medium supplemented with 10% fetal bovine serum (FBS), 2 mM L-glutamine, 100 U/ml penicillin and 100 μg/ml streptomycin, while all other cells were cultured in RPMI 1640-based medium in 5% CO_2_ environment at 37 °C.

### Reagents

Small molecule inhibitors of OGA PugNAc and thiamet G were obtained from Abcam (Cambridge, UK), while ketoconazole (KCZ)^12^ was from Crosschem Intercontinental Company, Derb & Co. (Lugano, Switzerland). *Cis*-diamminedichloroplatinum II (cisplatin, CDDP) was obtained from Sigma-Aldrich (St. Louis, MO). Hoechst 33342 and propidium iodide (PI) were obtained from Molecular Probes (Eugene, OR). Antibodies for *O*-GlcNAc and ubiquitin were obtained from Abcam, while antibody for p53 was from Santa Cruz Biotechnology (Dallas, Texas). All other antibodies and proteasome inhibitor MG132 were from Cell Signaling Technology (Beverly, MA).

### CRISPR guide RNA design, vector construction and lentivirus production

CRISPR/Cas9 system containing Cas9 nuclease (Addgene #52962, Cambridge, MA) and two gRNAs was used to induce transcriptional repression of *MGEA5*. Briefly, gRNAs targeting *MGEA5* (sequence #1: CACAGCCTCGCTCTCCGCTT and #2: CGCAAGCGCAGTGCGGATAAAC) were designed using CRISPR Design tool (http://crispr.mit.edu/) and cloned into human gRNA expression vector containing a mouse U6 promoter and a constitutive CMV promoter driving an *mCherry* gene (Addgene plasmid #44248)^36^, as described previously^37^. Lentivirus production was performed using HEK293T packaging cells (ATCC) in conjunction with pCMV.dR8.2 dvpr lentiviral packaging and pCMV-VSV-G envelope plasmids (Addgene plasmids #8454 and 8455)^38^. Cells were incubated with Cas9 and gRNA viral particles in the presence of hexadimethrine bromide (HBr) for 48 h. The transfection efficiency was determined by using an mCherry reporter and was found to be ~80%.

### Short hairpin RNA-mediated gene knockdown

Retroviral and lentiviral plasmids carrying short hairpin RNA sequences against human *TP53* and *MYC* were obtained from Addgene (plasmids #10672 and 29435)^39, 40^. Retrovirus production was performed using Platinum-A packaging cell lines and lentivirus production was performed using HEK293T packaging cells as described above. Cells were incubated with shp53 or shMyc viral particles in the presence of HBr for 36 h and p53 and c-Myc knockdown was analyzed prior to use by Western blotting.

### Plasmids and transfection

Control GFP and p53 plasmids were obtained from Invitrogen (Carlsbad, CA), while c-Myc plasmid was a gift from Wafik El-Diery (Addgene plasmid #16011)^41^. Briefly, 1 × 10^6^ cells were suspended in 100 μl nucleofection solution SF and transfected with 2 μg of plasmid by nucleofection using 4D Nucleofector^TM^ (Lonza, Cologne, Germany) with EH-158 device program. The transfected cells were checked for GFP fluorescence, and p53 and c-Myc expression levels were identified by Western blotting.

### Apoptosis assay

Apoptosis was determined by Hoechst 33342 assay and by cell diameter and DNA content analyses. In the Hoechst assay, cells were incubated with 10 μg/ml Hoechst 33342 for 30 min and analyzed for apoptosis by scoring the percentage of cells having condensed chromatin and/or fragmented nuclei by fluorescence microscopy (Eclipse Ti-U with NiS-Elements, Nikon, Tokyo, Japan). The apoptotic index was calculated as the percentage of cells with apoptotic nuclei over total number of cells. For DNA content analysis, cells were harvested, fixed with ice cold 70% ethanol overnight and stained with propidium iodide (PI) solution (50 μg/ml; Molecular Probes) containing 0.2% Tween 20 and 1 μg/ml RNase at room temperature for 30 min. PI fluorescence was analyzed using flow cytometry (BD LSRII; Becton Dickinson, Rutherford, NJ) at the excitation and emission wavelengths of 535 nm and 617 nm and fragmented DNA (sub-G0 cell cycle phase) was determined. Cell diameter was measured using an automated cell counter (Sceptor^TM^ 2.0, Merck Millipore, Billerica, MA), which is an impedance-based particle detection system employing the Coulter principle that enables the discrimination of apoptotic cells^42^.

### RNA isolation and RT-PCR

Total RNA was prepared using TRIzol reagent (Invitrogen) and cDNA was prepared using SuperScript III first-strand synthesis system and oligo (dT) primers (Invitrogen). qPCR analysis was carried out on a 7500 Fast real-time PCR using a Power SYBR Green PCR master mix (Applied Biosystems, Foster City, CA). Initial enzyme activation was performed at 95 °C for 10 min, followed by 40 cycles of denaturation at 95 °C for 15 sec and primer annealing/extension at 60 °C for 1 min. Relative expression of each gene was normalized against the housekeeping *GAPDH* gene product.

### Caspase-3 activity assay

Caspase activity was determined by detecting the cleavage of specific substrate DEVD-AFC using a commercial assay kit (Biovision, Milpitas, CA). After specific treatments, cell lysates were prepared and incubated with DEVD-AFC (50 μM) for 1 h. AFC fluorescence was measured using a fluorescence plate reader (Synergy H1, BioTek, Winooski, VT) at the 400-nm and 520-nm excitation and emission wavelengths. Caspase-3 activity was expressed as the ratio of signals from the treated and control samples.

### Western blot analysis

After specific treatments, cells were incubated in a commercial lysis buffer (Cell Signaling Technology) and a protease inhibitor mixture (Roche Molecular Biochemicals, Indianapolis, IN) at 4 °C for 30 min. Protein content was analyzed using BCA protein assay (Pierce Biotechnology, Rockford, IL) and 50 μg of proteins were resolved under denaturing conditions by SDS–PAGE and transferred onto PVDF membranes. Membranes were blocked with 5% nonfat dry milk, incubated with appropriate primary antibodies at 4 °C overnight, and subsequently incubated with peroxidase-conjugated secondary antibodies for 1 h at room temperature. The immune complexes were analyzed by enhanced chemiluminescence detection system on a digital imager (ImageQuant LAS, GE Healthcare, Pittsburgh, PA).

### Co-immunoprecipitation, ubiquitination, and *O*-GlcNAcylation

Cell lysates (200 μg protein) were immunoprecipitated using Dynabeads Protein G magnetic beads (Invitrogen). Briefly, the beads were conjugated with anti-p53 or anti-c-Myc antibody for 10 min at room temperature. The conjugated beads were then resuspended with cell lysates for 30 min at room temperature. The immune complexes were washed four times and resuspended in 2x Laemmli sample buffer. They were then separated by SDS-PAGE and analyzed for ubiquitination or *O*-GlcNAcylation using anti-ubiquitin or anti-*O*-GlcNAc (RL2) antibody respectively.

### Statistical analysis

The data represent means ± s.d. from three or more independent experiments as indicated. Statistical analysis was performed by Student’s *t*-test at a significance level of *p* < 0.05.

## Electronic supplementary material

Supplementary information
