# Supplementary material for: Hyper-O-GlcNAcylation induces cisplatin resistance via regulation of p53 and c-Myc in human lung carcinoma
Source: Sci Rep. 2017 Sep 6;7:10607. doi: 10.1038/s41598-017-10886-x (PMC5587763; doi:10.1038/s41598-017-10886-x)
Supplement: Supplementary file 1 — Supplementary information [file 41598_2017_10886_MOESM1_ESM.pdf]

Hyper-*O*-GlcNAcylation induces cisplatin resistance via regulation of p53 and c-Myc in human lung carcinoma

Sudjit Luanpitpong,[a] Paweorn Angsutararux,[a] Parinya Samart,[a,b] Nawin Chanthra,[a] Pithi Chanvorachote,[c] Surapol Issaragrisil[a,d,e,*]

[a]Siriraj Center of Excellence for Stem Cell Research, Faculty of Medicine Siriraj Hospital, Mahidol University, Bangkok 10700, Thailand

[b]Department of Immunology, Faculty of Medicine Siriraj Hospital, Mahidol University, Bangkok 10700, Thailand

[c]Department of Pharmacology and Physiology, Faculty of Pharmaceutical Sciences, Chulalongkorn University, Bangkok 10330, Thailand

[d]Division of Hematology, Department of Medicine, Faculty of Medicine Siriraj Hospital, Mahidol University, Bangkok 10700, Thailand

[e]Bangkok Hematology Center, Wattanaosoth Hospital, BDMS Center of Excellence for Cancer, Bangkok 10310, Thailand

***Correspondence:** Surapol Issaragrisil, MD, Department of Medicine, Faculty of Medicine Siriraj Hospital, Mahidol University, 2 Siriraj Hospital, Bangkoknoi, Bangkok 10700, Thailand; Tel. +66 2 419 4446; Email: surapol.iss@mahidol.ac.th.

Number of supplementary figures: 4

Supplementary information

Supplementary information includes Supplementary Figures S1−S4.

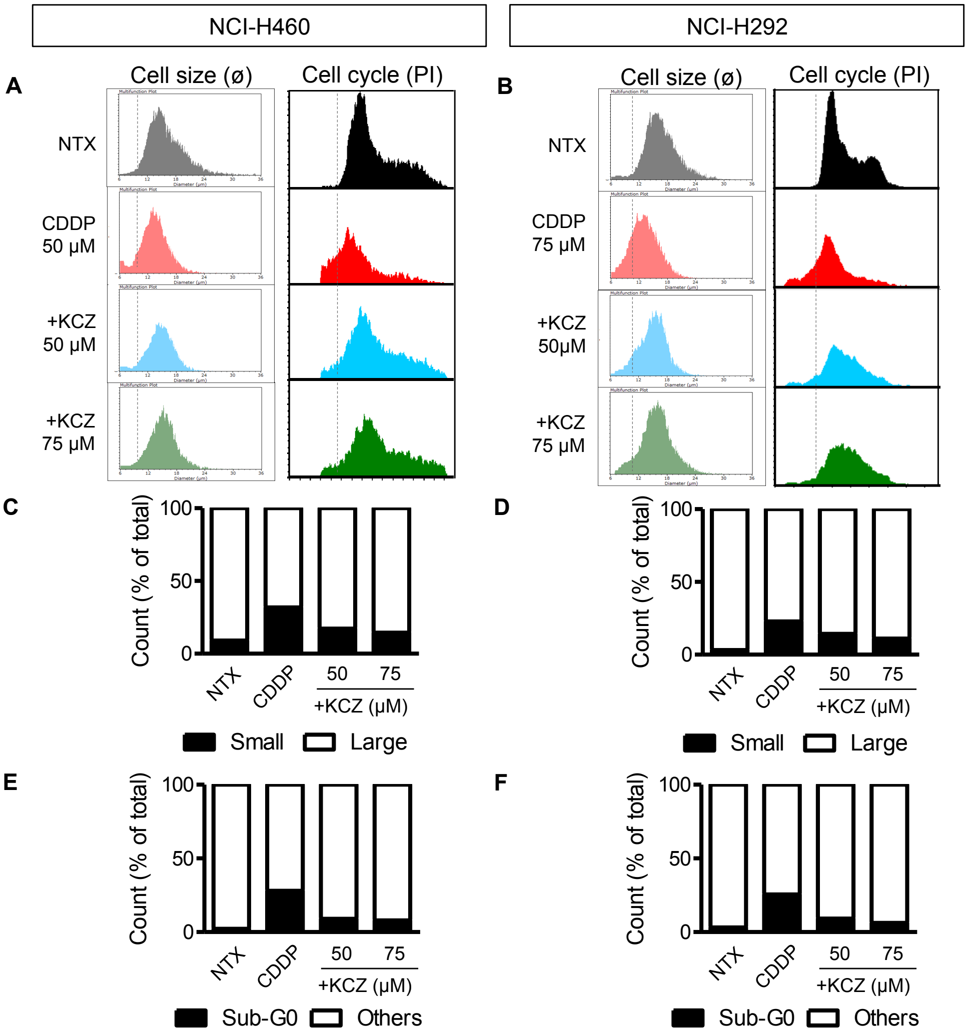

Supplementary Figure S1. Effects of *O*-GlcNAcase inhibitor on morphologic and biochemical hallmarks of cisplatin-induced apoptosis. (A, B) Human lung carcinoma NCI-H460 and NCI-H292 cells were co-treated with cisplatin (CDDP; 50 or 75 μM) and a highly selective *O*-GlcNAcase inhibitor ketoconazole (KCZ; 50−75 μM) for 24 h and analyzed for cell diameter using Scepter 2.0 cell counter and DNA content by flow cytometry using propidium iodide (PI) as a fluorescent probe. Representative histograms of cell size and DNA content with dash lines indicating cell distribution into (i) small or large cells, and (ii) sub-G_0 and other phases are shown. (C−F) Plots

are the proportions of cells in the small and large diameter fractions (C, D) and cells in sub-G_0 apoptotic phase and others (E, F).

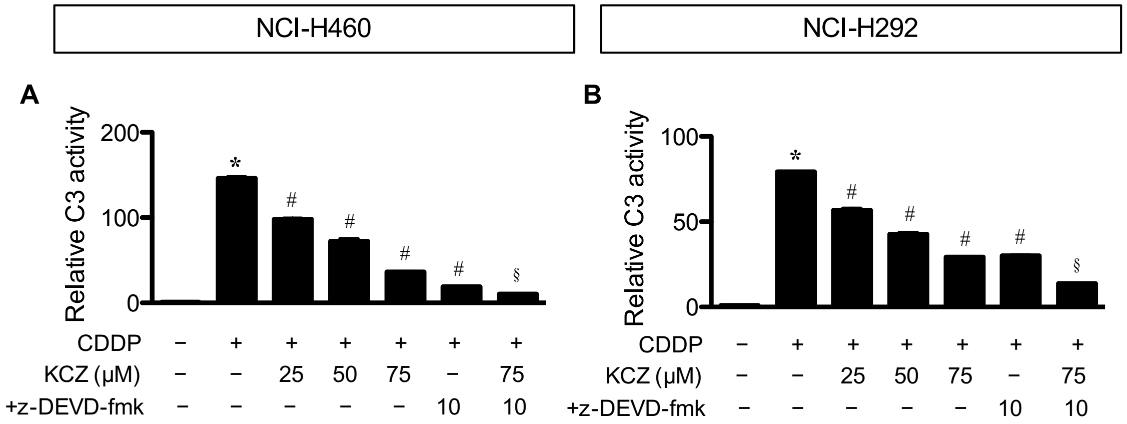

Supplementary Figure S2. Effect of *O*-GlcNAcase inhibitor on caspase-3 activation. (A, B) Human lung carcinoma NCI-H460 and NCI-H292 cells were co-treated with cisplatin (CDDP; 50 or 75 μM) and a highly selective *O*-GlcNAcase inhibitor ketoconazole (KCZ; 50−75 μM) with or without caspase inhibitor z-DEVD-fmk (10 μM), and cell lysates were prepared and measured for caspase-3 (C3) activity using fluorometric substrate DEVD-AFC. Plots are means ± S.D. ($n = 3$). *$p < 0.05$ versus non-treated control. $^{\#}p < 0.05$ versus CDDP-treated cells. $^{\S}p < 0.05$ versus CDDP-treated cells in the presence of KCZ. Plots are means ± S.D. ($n = 3$). *$p < 0.05$ versus non-treated control. $^{\#}p < 0.05$ versus CDDP-treated cells.

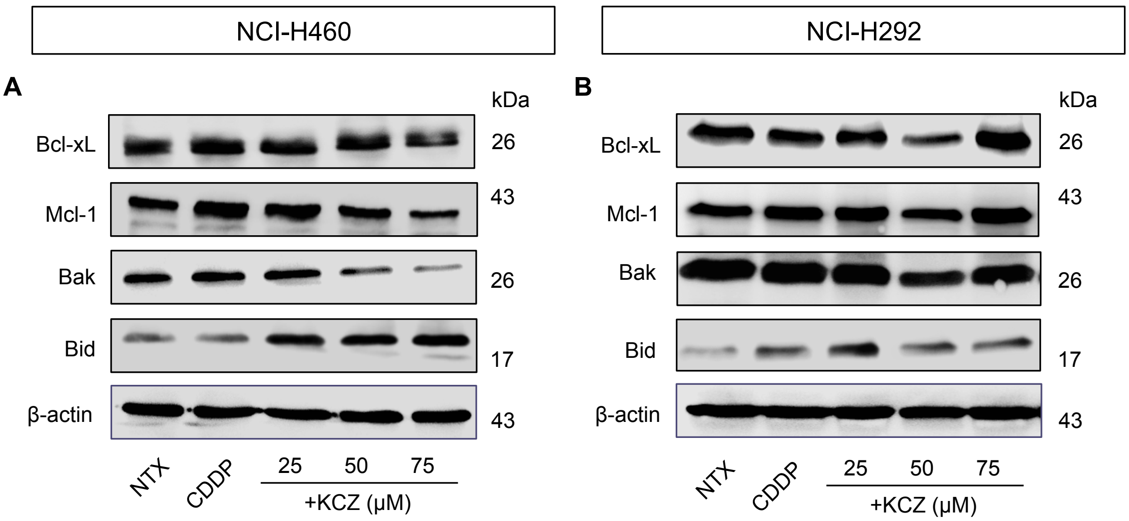

Supplementary Figure S3. Effects of *O*-GlcNAcase inhibitor on Bcl-2 family proteins. (A, B) Human lung carcinoma NCI-H460 and NCI-H292 cells were co-treated with cisplatin (CDDP; 50 or 75 μM) and *O*-GlcNAcase inhibitor ketoconazole (KCZ; 25−75 μM) for 24 h and cell lysates were prepared and analyzed for Bcl-xL, Mcl-1, Bak and Bid by Western blotting. Blots were reprobed with anti-β-actin antibody to confirm equal loading of the samples.

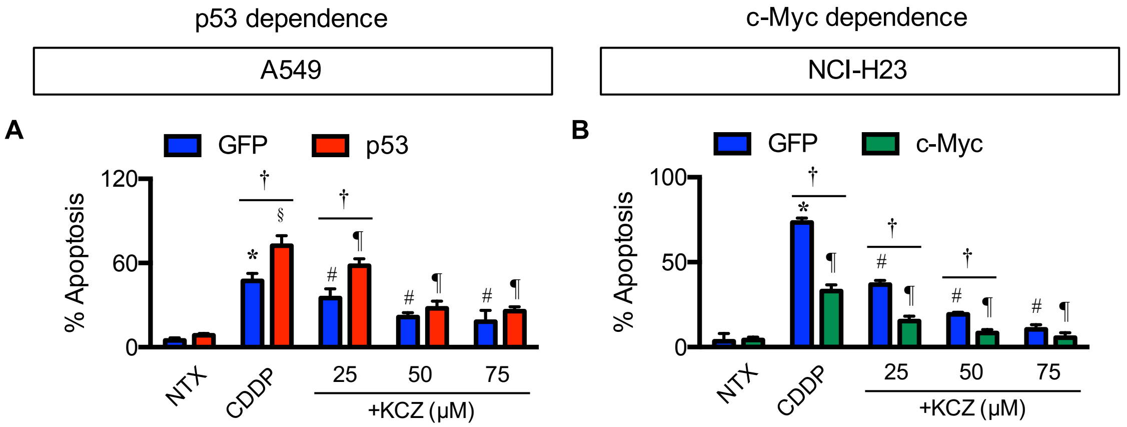

Supplementary Figure S4. Effects of p53 and c-Myc overexpression on cisplatin-induced apoptosis. (A, B) Human lung carcinoma A549 and NCI-H23 cells were transfected with p53 or

c-Myc expression plasmid, co-treated with cisplatin (CDDP) and ketoconazole (KCZ) for 24 h, and analyzed for apoptosis by Hoechst 33342 assay. Plots are means ± S.D. (n = 3). $^{*,\S}p < 0.05$ versus non-treated GFP or p53/c-Myc-overexpressed cells. $^{\#,\P}p < 0.05$ versus CDDP-treated GFP or p53/c-Myc-overexpressed cells. $^{\dagger}p < 0.05$ versus CDDP-treated GFP cells.
